# Infection control and tuberculosis among health care workers in Viet Nam, 2009-2013: a cross-sectional survey

**DOI:** 10.1186/s12879-016-1993-1

**Published:** 2016-11-10

**Authors:** Edine W. Tiemersma, Nguyen Thien Huong, Pham Hoang Yen, Bui Thi Tinh, Tran Thi Bich Thuy, Nguyen Van Hung, Nguyen Thanh Mai, Suzanne Verver, Agnes Gebhard, Nguyen Viet Nhung

**Affiliations:** 1 KNCV Tuberculosis Foundation, Benoordenhoutseweg 46, 2596 BC The Hague, The Netherlands; 2 KNCV Tuberculosis Foundation Vietnam Country Office, 130 Mai Anh Tuan Street, Hanoi, Vietnam; 3 National Tuberculosis Control Program of Vietnam, 463 Hoang Hoa Tham, Hanoi, Vietnam; 4 Pham Ngoc Thach Hospital, 120 Hong Bang, Ho Chi Minh City, Vietnam

**Keywords:** Tuberculosis, Health care workers, Viet Nam, Questionnaire, Infection control

## Abstract

**Background:**

Data on tuberculosis (TB) among health care workers (HCW) and TB infection control (TBIC) indicators are rarely available at national level. We assessed multi-year trends in notification data of TB among HCW and explored possible associations with TBIC indicators.

**Methods:**

Notified TB incidence among HCW and 3 other TBIC indicators were collected annually from all 64 provincial and 3 national TB facilities in Vietnam. Time trends in TB notification between 2009 and 2013 were assessed using linear regression analysis. Multivariate regression models were applied to assess associations between the facility-specific 5-year notification rate and TBIC indicators.

**Results:**

Forty-seven (70 %) of 67 facilities contributed data annually over five years; 15 reported at least one HCW with TB in 2009 compared to six in 2013. The TB notification rate dropped from 593 to 197 per 100,000 HCW (p_trend_ = 0.02).

Among 104 TB cases reported, 30 were employed at TB wards, 24 at other clinical wards, ten in the microbiology laboratory, six at the MDR-TB ward, and 34 in other positions.

The proportion of facilities with a TBIC plan and focal person remained relatively stable between 70 % and 84 %. The proportion of facilities providing personal protective equipment (PPE) to their staff increased over time. Facilities with a TBIC focal person were 7.6 times more likely to report any TB cases than facilities without a focal person.

**Conclusions:**

The TB notification rates among HCW seemed to decrease over time. Availability of PPE increased over the same period. Appointing a TBIC focal person was associated with reporting of TB cases among HCW. It remains unclear whether TBIC measures helped in reduction of the TB notification rates in HCW.

## Background

Since long, tuberculosis (TB) is regarded as an occupational risk for health care workers (HCW), and HCW have been shown to have a higher risk of active TB than the general population in multiple studies [1–4]. However, little is known about the burden of TB disease among HCW globally since TB among HCW is not registered as such in most routine TB surveillance systems. The World Health Organization (WHO) intends to include an indicator for monitoring of TB among HCW, which is the ratio of TB notification rate (all forms) in HCW (all staff) over the TB notification rate in the general population, adjusted for age and sex, in future Global TB reports [5].

One of the major objectives of the 2009 “WHO Policy on TB Infection Control in healthcare facilities, congregate settings and households” [6] is to establish effective TB infection control (IC) measures at healthcare facilities. Successful implementation of TBIC measures is important for preventing HCW, patients and visitors from becoming infected with drug sensitive and drug resistant TB [1–4, 7–9]. In addition, monitoring the occurrence of active TB among HCW could be a proxy for assessing reduced TB transmission associated with the implementation of TBIC in healthcare facilities [10].

Vietnam is one of the 22 high TB burden countries, with an estimated incidence rate of 144 per 100,000 per year (95 % uncertainty interval, 121-174) in 2013 [5]. To date, no studies have been conducted to assess the burden of active TB disease among HCW in the country, and the routine, patient based, electronic recording and reporting system does not record HCW specifically. However, one study comparing TB infection prevalence in one of the four referral hospitals with the adjacent non-TB hospital, showed a high prevalence of latent TB infection among HCW in the TB hospital (38 %-55 % in the youngest age group (20-29 years), depending on the method used), and a two times increased TB infection risk compared to HCW in the same age group in the non-TB hospital. This increased risk may have been due to inadequate IC measures: HCW and TB patients used the same (and only) hospital entrance, and only surgical masks, instead of N95 respirators, were available [11].

Vietnam is one of the first high TB incidence countries collecting annual data on TB among HCW and other TBIC indicators in all national and provincial TB and lung diseases facilities for at least five years. Here, we report the results of this annual assessment.

## Methods

### Aim

We aimed to assess time trends in the annual notification rates (2009-2013) of TB among HCW in these provincial and national facilities. Also, we explored potential associations between TB notification rates and different TBIC indicators reported.

### Design

To get better insight in the burden of TB among HCW employed in public TB facilities connected to the National TB Program (NTP) in Vietnam, NTP has developed four main TBIC indicators:

- Proportion of HCW with TB among all HCW per year;
- Proportion of health facilities that have a (valid, i.e., not outdated) TBIC plan;
- Proportion of health facilities that have an appointed TBIC focal person;
- Proportion of HCW working in the multidrug resistant (MDR)-TB ward or in the culture and/or drug susceptibility testing (DST) section of the hospital’s laboratory that is provided at least one N95 respirator per week.

Based on these indicators, a short questionnaire was designed in English and translated in Vietnamese. It included additional questions on the characteristics of the TB patients among HCW, the duration of validity of the TBIC plan, the presence of a TBIC focal person and provision of personal protective equipment (PPE) to HCW working in the culture and/or DST section of the microbiology department and HCW serving in MDR-TB wards. Annually, between 2010 and 2013, the form was sent to all national and provincial TB facilities, requesting the administration department of the hospital to complete the form. Information was collected on the period 2009-2013 by the TBIC focal person, who was usually part of the infection control committee overseeing implementation of IC policies in the hospital. Minor changes were made in the form over the years (see Results section).

### Setting

The provincial facilities for diagnosis and treatment of TB of all 63 provinces in Vietnam were included in this study: 43 TB and lung diseases hospitals, 18 preventive medicine centers for social diseases control and prevention, two preventive medicine centers, and one TB station. Hanoi both has a provincial TB hospital and a preventive medicine center for social diseases. Also, the three national TB and lung disease hospitals were included so that the total number of health facilities was 67. National hospitals are highly specialized hospitals that provide secondary and tertiary care to patients, most of whom have been referred from district and provincial levels, while provincial TB and lung disease hospitals usually provide secondary care and serve as referral centers for district TB units. The Ministry of Health’s circular 18/2009/TT-BYT, published on 14 October 2009, provided guidance in implementation of IC activities in health care facilities and led to the start of IC implementation in all TB services within NTP. Annual medical check-up for HCW is required by law, and involves mandatory X-ray for all those suspected of TB (i.e. with cough of more than two weeks). These check-ups are executed by medical examination facilities, which aggregate all cases diagnosed with any of 34 occupational diseases and report these twice a year to the Ministry of Health. TB is regarded occupational if active disease is found in a person working in a high-risk location (i.e., *M. tuberculosis* aerosols detected during periodic assessments) for a minimum duration of 6-12 months, depending on the type of TB (12 months for pulmonary, bone/joint, and urinogenital TB, 6 months for other forms of TB).

In Vietnam, Culture/DST rooms in the TB laboratories all use negative pressure. MDR-TB wards in principle use natural ventilation, but if there are less than 12 air changes per hour, natural ventilation is combined with an exhaust fan and ultraviolet germicidal irradiation (UVGI).

### Definitions

A health facility was defined as any TB service at provincial or supra-provincial level, as described under *Setting*.

A HCW was defined as anyone occurring on the payroll of the health facility in the specific year of reporting. This includes staff working in any clinical department, in paramedical sections (such as pharmacy), laboratories, and administrative and other support sections of the health facility.

TB was defined as active TB, and includes pulmonary (new and retreatment) and extra-pulmonary TB. Since reporting was anonymous we could not check whether these patients were notified and/or bacteriologically confirmed, and included any patient reported by the facility.

A TBIC plan is a written protocol for the prompt recognition, separation, provision of services, investigation for TB and referral of patients with suspected or confirmed TB disease in the health facility [12]. The TBIC plan was considered valid if the validity period included the reporting period. Since the TBIC plans were not available to the researchers, no content checks were conducted.

A TBIC focal person is the person in the health facility who is responsible for coordination of all TBIC activities in the health facility.

### Ethical issues

The project was approved by the Research Board of the National Lung Hospital in Hanoi. Only data that had been routinely collected by each health facility were requested. Data on HCW were collected in an aggregated manner. No personal information about HCW was requested. Therefore, no personal informed consent was obtained. All health facilities received a code before analysis. All data were analyzed in such a way that the information presented does not provide sufficient information for the identification of persons.

### Data entry and analysis

#### Data entry and validation

Submitted forms were checked for completeness and consistencies. Inconsistencies and omissions were checked and solved by phone and email with the health facility’s contact person. All data were entered in a pre-designed EpiData data entry sheet (www.epidata.dk). The entered data were compared with the paper forms by a person who had not been involved in data entry.

Data analysis was performed in Stata/SE 11.1 for Windows (Stata Corp., College Station, Texas, USA; www.stata.com).

### Data analysis

Notification rates were calculated by dividing the summed number of HCW with TB disease in a specific year by the summed number of HCW working in the health facilities in the same year. It was assumed that each HCW worked in the health facility for a full year. The rates were multiplied by 100,000 to obtain a notification rate per 100,000 HCW per year. Exact binomial 95 % confidence intervals (CIs) were calculated around each annual rate. Time trends were assessed applying random effects negative binomial regression analysis on the annual notification rates between 2009 and 2013 after specifying the data as panel (time series) data.

Health facilities were grouped to assess health facility characteristics as follows: (national) referral centers for TB (*n* = 4; including the three national TB hospitals and one provincial TB hospital acting as a tertiary referral center), provincial TB hospitals (*n* = 43; including all but one provincial hospitals for TB and lung diseases), and provincial preventive centers (*n* = 20; including 18 preventive medicine center for social diseases control and prevention and two TB stations).

**Fig. 1:**
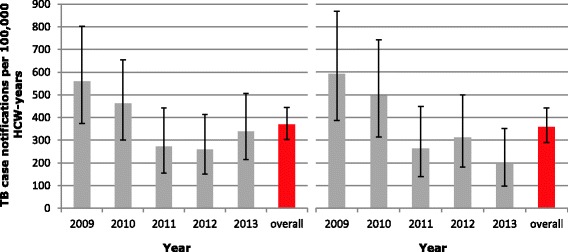
Reported annual TB case notification rate per 100,000 health care worker (HCW) years. Left panel includes all health facilities providing information on the number of TB cases and the number of HCW in any given year (2009-2013); right panel includes only the health facilities reporting this information for all five years. Error bars depict 95 % confidence intervals

Multivariate regression models were applied to assess the association between the health facility specific 5-year notification rate and health facility characteristics. Because of the (bimodal) distribution characteristics of the 5-year TB notification rates (see Fig. 1), different multivariate regression models were applied. First, a backward stepwise logistic regression model was built in which the dependent variable was either 1 (any TB cases reported between 2009 and 2013) or 0 (no TB cases at all between 2009 and 2013) to account for the 42 % of health facilities reporting no cases between 2009 and 2013. All variables for which the p-value was less than 0.2 were kept in the model. To assess the associations between notification rates and facility characteristics in those facilities from which cases were reported, the TB notification rate was first log-transformed and this transformed notification rate was analyzed using simple linear regression models. Here, backward stepwise linear regression was applied leaving all variables for which the *p*-value was less than 0.2 in the model. Time trends were calculated in- and excluding health facilities that did not provide data for all of the years 2009-2013. A *p*-value smaller than 0.05 was regarded as statistically significant.

## Results

### Tuberculosis notification rates among health care workers

The number of health facilities responding to the survey varied between 57/67 (85 %) in 2012 and 2013 and 64/67 (96 %) in 2011 (Table 1). The number of health facilities reporting at least one TB case for the respective year of reporting decreased between 2009 and 2013: from 18/61 (30 %) to 9/54 (17 %) (*p* = 0.02).

**Table 1:** Tuberculosis (TB) cases among health care workers in provincial and national TB facilities, 2009-2013^a^

Characteristic	Year of report					Total
	2009	2010	2011	2012	2013	
Number of health facilities sending report	62	63	64	57	57	
Number of HCW working in these facilities	5,225	5,455	5,940	6,580	6,811	
Number of health facilities reporting the number of TB cases among HCW	61	61	62	54	54	
Number of HCW in facilities reporting the number of TB cases	5,183	5,396	5,870	6,580	6,811	29,840
Number of facilities reporting at least one TB case	18	18	12	13	9	39^b^
Number of TB cases (all forms)	29	25	16	17^c^	23	110
Notification rate of all TB/100,000 HCW-years	560	463	273	258	338	369
95 % confidence interval	375-803	300-684	156-443	151-414	214-507	303-444
*Among health facilities reporting data for 5 consecutive years (n = 47)*						
Number of HCW working in these facilities	4,382	4,648	4,954	5,453	5,594	25,031
Number of facilities reporting at least one TB case	15	16	10	12	6	30^b^
Number of TB cases (all forms)	26	23	13	17	11	90
Notification rate of all TB/100,000 HCW-years	593	495	262	312	197	360
95 % confidence interval	388-868	314-742	140-448	182-499	98-352	289-442
Number of facilities with complete data for type of TB	61	60	62	52	52	
Number of TB cases in these facilities	29	24	16	15	17	101 (92 %)
Number of TB cases by type of TB:						
New pulmonary TB	19	10	9	8	11	57 (56 %)
Previously treated pulmonary TB	3	1	1	1	0	6 (6 %)
Extra-pulmonary TB	7	13	6	6	6	38 (38 %)
Number facilities with complete data for job category of HCW with TB	61	61	62	53	54	
Number of TB cases in these facilities	27	24	15	16	22	104 (95 %)
Number of TB cases among HCW employed in/at:						
microbiology department^d^	6	1	2	0	1	10 (10 %)
TB wards	8	8	2	6	6	30 (29 %)
MDR-TB ward^d^	1	2	2	1	0	6 (6 %)
other (non-TB) clinical wards	3	4	2	3	12	24 (23 %)
other positions	9	9	7	6	3	34 (33 %)

^a^Abbreviations used: *NTP* national tuberculosis program, *TB* tuberculosis, *HCW* health care worker(s), *MDR* multidrug resistant, ^b^Annual numbers do not sum up to the total as some health facilities reported at least one TB case in more than one of the five years; ^c^One provincial TB hospital was excluded since one TB case was reported for 2012 without providing the number of health care workers in that year, so that notification rate cannot be calculated; ^d^in total, 26 facilities had a culture laboratory and 25 hospitals reported having an MDR-TB ward

The notification rate of all types of TB dropped from 559 per 100,000 HCW per year in 2009 to 338 per 100,000 HCW per year in 2013 (Fig. 1, left panel).

A time trend in TB notification rates was calculated using data from 47 facilities that reported data for all five years. In these facilities, the notification rates dropped significantly from 593 (95%CI, 388-869) per 100,000 HCW years in 2009 to 197 (95%CI, 98-352) per 100,000 HCW years in 2013, (*p* = 0.02; beta-coefficient -0,2, 95%CI, -0.36 to -0.03; Fig. 1, right panel). We repeated this analysis excluding four health facilities with a reported increase of >100 % in health staff within one year. This did not change the results: the decrease remained statistically significant (*p* = 0.04; data not shown).

Overall, 56 % of the reported TB cases were registered new pulmonary TB cases, 6 % were previously treated pulmonary TB cases, and 38 % were registered with extra-pulmonary TB. The distribution remained more or less stable over the years.

Of a total of 104 (95 %) TB cases for whom the working position was recorded, 30 (29 %) were reported among staff employed at TB wards, 24 (23 %) among staff from other (non-TB) clinical wards, 10 (10 %) among microbiology laboratory staff, six (6 %) among staff working at the MDR-TB ward, and 34 (33 %) among staff working in other positions. It remains unknown how the number of TB cases reported relates to the number of staff members per working position, as staff counts per working position were not included in the data collection form for feasibility reasons.

Over the years, especially among workers in the microbiology departments a reduction of TB notification rates was seen (beta-coefficient -0.61 (95 % CI -1.18 to -0.03), *p* = 0.04).

### TBIC measures and TB among health care workers

In the period of 2009-2011, 48 of 64 health facilities (75 %) reported having a TBIC plan available; this plan was reported to be available by 45 of 56 facilities (80 %) reporting over the period in 2012-2013 (Table 2).

**Table 2:** TBIC indicators reported by provincial and national TB facilities to the NTP, 2009-2013^a^

Indicator	Number of facilities	Period of reporting		
		2009-2011	2012-2013	Total (with information/answer ‘yes’ for both periods)
TBIC plan				
	Number (%) with information	64	56	53
	Number (%) yes	48 (75 %)	45 (80 %)	37 (70 %)
TBIC focal person				
	Number (%) with information	63	56	52
	Number (%) yes	52 (83 %)	47 (84 %)	38 (73 %)
PPE provided to staff				
	Number with TB culture lab	23	24	20
	Number of those answering the question	23	21	19
	Number (%) of those with PPE	14 (61 %)	19 (90 %)	11 (55 %)
	Number with MDR-TB ward	15	24	14
	Number of those answering the question	15	22	12
	Number (%) of those with PPE	10 (67 %)	22 (100 %)	9 (64 %)

^a^Abbreviations used: *TB* tuberculosis, *IC* infection control, *NTP* national tuberculosis program, *MDR* multidrug resistant, *PPE* personal protective equipment

In a sensitivity analysis, taking only those health facilities into account reporting on this indicator both in the period 2009-2011 and 2012-2013 (*n* = 53), the proportion of facilities with a TBIC plan also remained stable (41 (77 %) had a plan in 2009-2011 and 43 (81 %) had one in 2012-2013). The median period of validity of a TBIC plan was three years (interquartile range, 2-5 years), with a maximum of 13 years.

It is not known which TBIC plans had been based on (inter)national guidelines, and how many of these had been made with input from TBIC experts.

The proportion of facilities reporting to have an appointed TBIC focal person was 83 % (52/63 facilities) for 2009-2011 and 84 % (47/56 facilities) for 2012-2013, and 73 % taking only the 52 health facilities into account that reported on this indicator for both periods (Table 2).

N95 respirators were provided in the majority of microbiology laboratories providing culture (with or without DST) and health facilities with an MDR-TB ward (Table 2). The proportion of such facilities having PPE available for their staff increased between 2009-2011 and 2012-2013 (from 61 % to 90 %, *p* = 0.02 for culture/DST laboratories, and from 67 % to 100 %, *p* = 0.01 for MDR-TB wards), assuming those reporting are representative for all facilities with an MDR-TB ward and/or a culture/DST laboratory.

Only six health facilities had not implemented any TBIC measures over the reporting period; these include one TB unit in a provincial hospital and five preventive medicine centers. Together, these facilities reported only one extra-pulmonary TB case in five years’ time. Having only limited data on TBIC measures available, we did not find an effect of any improvements in TBIC measures (defined as newly implementing a TBIC plan or appointing a TBIC person or starting the use of PPE in MDR-TB wards and culture laboratory sections) on the TB notification rates (data not shown). Among the facilities with MDR-TB wards and/or culture sections, the 5-year notification rates seemed lower in those with reported provision of N95 respirators (354/100,000 HCW-years, 95 % CI 277-445) than in those without (448/100,000 HCW-years, 95 % CI 180-921), but this difference was not statistically significant.

The notification rates were lower in those facilities that had no TBIC focal person compared to facilities with a TBIC focal person (Kruskal-Wallis ranksum test *p*-value, *p* = 0.08 for 2009-2011 and *p* = 0.04 for 2012-2013).

### Health facility characteristics and TB among health care workers

Table 3 presents notification rates by facility type and geographical zone (all reports of the health facilities received from 2009-2013 included). The notification rate was near-to-significantly higher in the referral centers compared to provincial health facilities (*p* = 0.06), with a conditional maximum likelihood estimate (CMLE) of the rate ratio of 1.5 (95 % CI 0.99-2.2). The TB notification rate in Southern health facilities was higher than in Northern, and statistically significantly higher than in Central facilities (*p* = 0.002; CMLE rate ratio 4.9, 95 % CI 1.7-20). The notification rate in the North was also higher than in the Central zone (*p* = 0.02; CMLE rate ratio 3.7, 95 % CI 1.3-14.7) (Table 3).

**Table 3:** Notification rate of all types of tuberculosis per 100,000 health care worker-years, 2009 – 2013^a^

Type of facility	Number of facilities^b^	Number of HCW-years	Number of TB cases^b^	Tuberculosis notifications, all types, per 100,000 HCW-years	
				Rate	(95 % CI)^c^
Type of facility					
Referral hospitals for TB & lung diseases	4	8,257	40	484	(346 - 660)
Provincial hospitals & centers for TB & lung diseases	42	18,388	60	326	(249 - 420)
Provincial preventive centers^d^	20	3,153	10	317	(152 - 583)
Region					
North	32	17,626	63	357	(275 - 457)
Central	12	3,068	3	98	(20 - 286)
South	22	9,157	44	481	(349 - 645)

^a^Abbreviations used in this Table: *TB* tuberculosis, *HCW* health care worker, *CI* confidence interval. ^b^One provincial TB hospital in Central Vietnam was excluded since one TB case was reported for 2012 without providing the number of health care workers in that year, so that notification rate cannot be calculated. ^c^95 % binomial exact confidence intervals are displayed. ^d^These include centers for social disease prevention (*n* = 18) and medical preventive centers (*n* = 2)

**Fig. 2:**
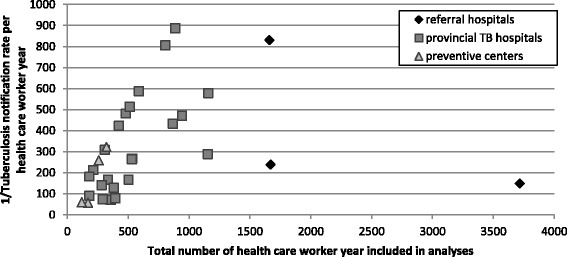
Five-year health care worker (HCW) TB notification rate versus number of HCW employed. Each marker depicts one health facility; the different types of markers refer to the different types of health facilities included

Among the provincial TB hospitals, there was a significant inverse linear association between the number of HCW employed and 5-year TB notification rate (*p* = 0.001; β-coefficient = 0.8, standard error = 0.2; Fig. 2).

Facilities with an appointed IC focal person were 7.6 times more likely to report any TB cases than facilities that had no focal person (Table 4). Compared to health facilities in Central Vietnam, facilities in North Vietnam were 10 times more likely to report a TB case (Table 4).

**Table 4:** Association between health facility characteristics (2013) and ≥1 tuberculosis case reported between 2009 and 2013

Characteristic	Univariate		Multivariate	
	Odds Ratio	95 % Confidence Interval	Odds Ratio	95 % Confidence Interval
Type of health facility				
Policlinic only	1	REF		
Additional inpatient treatment	6.9	2.1 - 23.1		
HCW-years (increase with 50 HCW-years^a^)	1.3	1.1 - 1.5	1.1	0.96 - 1.4
Location of the facility in:				
Central Vietnam	1	REF	1	REF
North Vietnam	12.0	2.5 - 58.5	10.0	1.6 - 61.2
South Vietnam	2.5	0.5 - 11.8	4.4	0.8 - 24.6
Presence of culture laboratory	2.7	0.9 - 7.9		
Presence of MDR-TB ward	2.7	0.9 - 7.9^b^		
(TB)-IC plan present	3.8	1.1 - 12.8		
Appointed focal person for (TB)-IC available	8.6	1.7 - 44.8	7.6	1.2 - 47.5
PPE available in culture labs/MDR-TB ward	3.7	1.2 - 11.1		

* Abbreviations used: *HCW* health care worker, *IC* infection control, *MDR* multidrug resistant, *PPE* personal protective equipment. The analyses in this table include 64/67 ﻿facilities, as 3 did not report sufficient data on TB cases; ^a^This can also be interpreted as an increase with 50 health care workers for each of the 5 years; ^b^This is not an erroneous duplication of the Odds ratio and confidence interval listed for presence of culture laboratory. Included were 25 centres with an MDR-TB ward and 25 centres with a culture laboratory (18 of which had both); 7 centres had no MDR-TB ward but a culture laboratory; 7 other centres had no culture laboratory but had an MDR-TB ward

## Discussion

### Main results of the study

Over the reporting period (2009-2013), the rate of all types of TB reported dropped from 559 per to 338 per 100,000 HCW-years. Including only the 47 health facilities reporting for five consecutive years, the notification rates dropped from 593 to 197 per 100,000 HCW years (test for trend, *p* = 0.02). While this is a marked decline, in 2013 the notification rate among HCW was still almost twice as high as in the general population (which was 111/100,000 in 2013 [5]). This may in part reflect better access to screening and awareness about the occupational risk of TB transmission, but will also reflect the higher risk of getting TB among HCW, which is consistently reported in almost all published literature on this topic [1, 2, 4, 13]. The most recent systematic review reported a relative risk (RR) of active TB disease for HCW compared to the general population of 3.7 (95 % CI 2.9-4.5) with RRs ranging from 1.2 to 14.7 depending on the setting [1]. It should be noted however that these RRs were not corrected for age and sex, since this information is often not available. Between 2009 and 2013, there was a statistically significant decline (*p* = 0.04) in the TB notification rates among staff working in TB laboratories. This decline may be the result of increased protection and awareness of staff, the availability of biosafety cabinets and improved laboratory practices as a result of continued training activities. Though a decline was also seen among staff working in MDR-TB departments, this was much weaker and failed to reach statistical significance. Notably, the proportion of health facilities providing PPE to staff increased from 61 % in 2011 to 79 % in 2013 for staff in MDR-TB wards (*p* = 0.17) and from 67 % to 92 % (*p* = 0.048) for staff in the culture/DST section of the microbiology laboratories. It should be noted that the number of TB patients found in non-TB related departments remained relatively high, and this is in line with results found by an earlier review [2]. Also it should be noted that the available data (all age/sex classes) shows no decline over time in notification rates among the general population during the study period.

Only a few studies have measured the effect of TBIC measures on the TB burden among HCW [7, 8, 10, 14–16] and from three of these studies, a decrease in latent TB infection was reported, but not a decrease in TB disease. One cross-sectional study only reported about TB disease [10]. A weak association was found between reported IC measures (especially environmental measures) and TB disease incidence, but it disappeared in the multivariable analysis. About 75 % of health facilities included in our report had a TBIC plan and an appointed TBIC focal person. These proportions remained stable over time. Presence of a TBIC focal person in the facility generally was associated with a higher probability of reporting any TB cases (adjusted Odds Ratio = 7.6 95 % CI 1.2-47.7). Similarly, presence of a TBIC plan was associated with finding one or more TB-cases among staff members. This may be the effect of less active case finding and reporting from facilities without a TBIC focal person as compared to facilities with a TBIC focal person. The alternative explanation may be that those facilities with more TB cases among HCW may be encouraged to appoint a TBIC focal person (reverse causality). The information that we collected was probably not specific enough to detect any effect of TBIC measures. Further, confidence intervals are wide since we conducted ecological analysis, with one data point for each health facility. Presence of a TBIC plan alone likely does not contribute to reduced transmission of TB or increased case finding within a facility. This information was not available from the routine reports unfortunately and additional data should be collected about TBIC practices.

Although there is evidence that implementation of IC measures reduces the burden of TB [2, 4], few studies evaluated the impact of TBIC measures in low and middle income countries (LMIC). One study found no effect on TB disease incidence [14] and two showed a reduction in tuberculin skin test conversion rates in HCW [7, 8, 15]. In LMIC, even low-cost strategies are rarely implemented [3, 15, 17] though recently IC has gained more attention and more is being done to protect HCW from TB infection and disease.

Not surprisingly, larger facilities (in terms of number of HCW) were more likely to report any TB cases. However, for those facilities in which TB cases were notified, the notification rates decreased with increasing facility size (rate ratio for an increase in size with 50 HCW-years: 0.94, *p* = 0.01). This decline in notification rates may be because many of the larger hospitals are tertiary care facilities which tend to pay more attention to TBIC measures, as national implementation of such measures usually starts in tertiary care hospitals which also have more resources available than other hospitals.

Provincial TB hospitals reported lower TB notification rates (329/100,000 HCW-years) when compared to other types of facilities (notably national/regional hospitals with a notification rate of 484/100,000 HCW-years). This may be explained by presence of more difficult-to-treat TB patients and conduct of higher-risk procedures in the larger hospitals, such as bronchoalveolar lavage and pneumectomy, and culture and DST in laboratories, but also by better awareness among HCW about the risks of TB transmission leading to better adherence to annual screening procedures, and more resources facilitating reporting of TB cases. However, more data are needed to learn which of these explanations contributes most.

The probability of reporting any TB cases was lower in Central than in North Vietnam (where two of the three regional and the national TB hospital are located). This association may reflect the underlying TB burden in the general population. The TB prevalence survey found a lower prevalence in Central Vietnam as compared to North and South Vietnam (*p* < 0.05) [18], and the data also match the overall TB notification data of NTP [19].

### Limitations

The data presented in this report was collected routinely from all provincial and supra-provincial facilities reporting to the NTP in Vietnam. To our knowledge, this information is rather unique and Vietnam is one of the few countries in the region collecting any indicator data on TBIC practices and on TB cases among HCW. However, routine collection of data on just a few indicators understandably has its limitations as much less information can be collected than is usually done in research settings.

First of all, this concerns indicators aggregated by health facility and data can thus not be linked to individual characteristics such as sex, age, working experience, and job type as has been done by others [10]. Also, comparison with the notification rates in the general population was impossible since the age and sex composition of our study population was unknown. Local laws prescribe aggregate data collection of TB among HCW. Moreover, TB is highly stigmatized in Vietnam, also among HCW, and therefore it was considered inappropriate to collect details that could lead to identification of HCW.

The data only include TB cases known to the health facility administration. In Vietnam, TB is still stigmatized [20] and this may cause HCW to seek diagnosis and treatment elsewhere. On the other hand, annual health screening is mandatory and includes chest X-ray imaging. It is known that X-ray screening is a sensitive method to detect pulmonary TB, also in the absence of TB symptoms [21]. This mandatory system also implies that these notification data were derived from active case finding, which is not comparable to the passive case finding strategies used in Vietnam to diagnose TB.

TB infection (measured by tuberculin skin test or Quantiferon test-conversion) better reflects recent transmission of *Mycobacterium tuberculosis* than TB disease notification since progression to TB disease may take several years and depends on other factors such as immune status of the host [22]. It may take time until changes in transmission will be measurable as changes in notification. Besides that, in countries with a generalized TB epidemic, transmission is likely to occur also in community settings [23–25].

We analyzed reported information that was not checked by the researchers on site. For example, we did inquire whether a TBIC plan and a focal person were available in the health center and whether N95 respirators were being distributed to the staff. However, though this provided some information, this does not necessarily mean that plans are implemented as they should and respirators are (always) used appropriately when guidelines prescribe so, especially if appropriate training on the importance of TBIC and its practical implementation is lacking.

This effort is a repetitive, low-workload activity, recording information on a minimum number of TBIC indicators, meeting the WHO recommendation to specifically include notifications of TB among HCW. The minimum dataset did not include the number of staff and TB patients in each job category, completeness of HCW screening and notification, occurrence of MDR-TB, and whether it concerned nosocomial or community-based transmission. To enable assessment of the implementation of TBIC measures, routine on-site monitoring is needed. To help countries set up a standard routine TB monitoring system among HCW a guide was developed by an international group of experts under TB CARE I [26]. To assess the effect of TB IC measures on nosocomial TB transmission, sophisticated studies would be needed, including careful assessment of potential epidemiological links and genotyping of TB strains isolated from patients using advanced molecular typing methods, preferably whole-genome sequencing [27].

Finally, presently there are no standardized ways for measuring occupational risk of TB infection and disease in high TB incidence populations (such as the Vietnamese population), or for assessing the implementation of TBIC measures [10]. Repeated surveys could be used to measure and compare infection rates in healthcare workers [28].

## Conclusion

Though the notification rate among HCW had dropped significantly from 593/100,000 in 2009 to 197/100,000*y in 2013, TB notification rates in public TB facilities were almost twice as high as in the general population (111/100,000*y; *p* = 0.02). There was a decline in the proportion of TB cases among HCW occurring among those employed in TB sections of the health facility. Around 80 % of the 67 health facilities included had a TBIC plan and a TBIC focal person. Facilities with a TBIC focal person and/or a TBIC plan were more likely to report any TB cases than facilities without such a person.

Vietnam is one of the few countries in the developing world that has set up this routine reporting of TB among HCW and thereby shows that occupational risks of TB transmission to HCW are taken seriously. Although data on TBIC indicators is now routinely collected, more detailed information is needed to assess risk factors for TB disease among HCW and the impact of TBIC measures. Since TB incidence among HCW is higher than that in the general position, implementation of TBIC measures needs continued attention.

## Abbreviations

CI: Confidence interval
CMLE: Conditional maximum likelihood estimate
DST: Drug susceptibility testing
HCW: Health care worker(s)
IC: Infection control
LMIC: Low- and middle income countries
MDR: Multidrug resistant
NTP: National tuberculosis program
PPE: Personal protective equipment
RR: Relative risk
TB: Tuberculosis
TBIC: Tuberculosis infection control
UVGI: Ultraviolet germicidal irradiation
WHO: World Health Organization
